# Unravelling the impact of insecticide-treated bed nets on childhood malaria in Malawi

**DOI:** 10.1186/s12936-023-04448-y

**Published:** 2023-01-13

**Authors:** Julie-Anne A. Tangena, Donnie Mategula, Luigi Sedda, Peter M. Atkinson

**Affiliations:** 1 Vector Biology Department, Liverpool School of Tropical Medicine, Liverpool, UK; 2 Malawi-Liverpool Wellcome Trust Clinical Research Programme, Blantyre, Malawi; 3 Lancaster Ecology and Epidemiology Group, Lancaster University, Lancaster, UK; 4 Lancaster Environment Centre, Lancaster University, Bailrigg, Lancaster, LA1 4YR UK; 5 Geography and Environmental Science, University of Southampton, Highfield, Southampton, SO17 1BJ UK; 6 Institute of Geographic Sciences and Natural Resources Research, Chinese Academy of Sciences, 11A Datun Road, Beijing, 100101 China

**Keywords:** Malaria indicator survey, Malawi, Insecticide-treated bed net, Bed net use

## Abstract

**Background:**

To achieve malaria elimination it is essential to understand the impact of insecticide-treated net (ITNs) programmes. Here, the impact of ITN access and use on malaria prevalence in children in Malawi was investigated using Malaria Indicator Survey (MIS) data.

**Methods:**

MIS data from 2012, 2014 and 2017 were used to investigate the relationship between malaria prevalence in children (6–59 months) and ITN use. Generalized linear modelling (GLM), geostatistical mixed regression modelling and non-stationary GLM were undertaken to evaluate trends, spatial patterns and local dynamics, respectively.

**Results:**

Malaria prevalence in Malawi was 27.1% (95% CI 23.1–31.2%) in 2012 and similar in both 2014 (32.1%, 95% CI 25.5–38.7) and 2017 (23.9%, 95% CI 20.3–27.4%). ITN coverage and use increased during the same time period, with household ITN access growing from 19.0% (95% CI 15.6–22.3%) of households with at least 1 ITN for every 2 people sleeping in the house the night before to 41.7% (95% CI 39.1–44.4%) and ITN use from 41.1% (95% CI 37.3–44.9%) of the population sleeping under an ITN the previous night to 57.4% (95% CI 55.0–59.9%). Both the geostatistical and non-stationary GLM regression models showed child malaria prevalence had a negative association with ITN population access and a positive association with ITN use although affected by large uncertainties. The non-stationary GLM highlighted the spatital heterogeneity in the relationship between childhood malaria and ITN dynamics across the country.

**Conclusion:**

Malaria prevalence in children under five had a negative association with ITN population access and a positive association with ITN use, with spatial heterogeneity in these relationships across Malawi. This study presents an important modelling approach that allows malaria control programmes to spatially disentangle the impact of interventions on malaria cases.

**Supplementary Information:**

The online version contains supplementary material available at 10.1186/s12936-023-04448-y.

## Background

Malaria is one of the most important causes of morbidity and mortality in Malawi. Since 2007, mass distribution of insecticide-treated nets (ITNs) to the demographically most vulnerable population groups has been a major part of Malawi’s vector control efforts. This, in combination with improved diagnosis and treatment of cases, has resulted in a 36% reduction in malaria cases, from an estimated 5.6 million cases in 2010 to 3.6 million in 2016 [1]. Despite this progress, continued investment in malaria control has not led to a further decrease in cases [1, 2] with an estimated 4.3 million cases still occurring in 2020.

The Malawi National Malaria Control Programme (NMCP) aimed to reduce malaria incidence by at least 50% from a 2016 baseline of 386 per 1000 population to 193 per 1000, and reduce malaria deaths by at least 50% from 23 per 100,000 population to 12 per 100,000 population by 2022 [3]. To reach these targets, it is necessary to understand the impact of current control activities and tailor future vector control to local epidemiological and entomological dynamics [4] in the context of a fast growing population. The Malawian population has grown almost 30% during from 2012 to 2022 to 20.4 million [5]. To control the malaria vectors, in 2007, the government started distributing long-lasting insecticide-treated nets (LLINs). It is generally accepted that ITN coverage has helped decrease malaria prevalence in Malawi [6–9]. However, retrospective studies have also found that a 13% increase in bed net access from 2012 to 2014 was not associated with a reduction in malaria burden in children [10]. This was corroborated by a similar study investigating overall trends within the 2012 and 2014 malaria indicator survey (MIS) data [11]. Comparison of data from 2004 and 2016 also showed that community malaria prevalence was not related to ITN use [12]. No personal, and limited community, protection from ITNs was found in a field study from 2012 [13]. These inconsistent conclusions suggest that ITN access and use may have a heterogeneous impact on malaria prevalence.

One of the main challenges for malaria elimination is the heterogeneity of the current malaria landscape [1, 14, 15]. It is not well understood why this heterogeneity has emerged, although the possible varied efficacy of ITNs could be partly responsible. ITNs can impact areas differently due to different vector population compositions and behaviours, climate variation and the presence of resistance [16–18]. Furthermore, gaps may exist between policy and implementation [19], with human behaviour one of the most complex variables involved in malaria transmission. The local population might accept, but not use and maintain nets, use nets for other purposes or migrate to areas with higher malaria risk [20–22]. Further reasons include social factors, such as autonomy in health care decisions [23], bed net integrity and insecticide degradation [24].

The best method for measuring the efficacy of ITNs directly is randomized controlled trials, ideally conducted across different parts of a country. No such randomized control trials have been conducted in Malawi due to the unethical nature of withholding nets from a proportion of the population. However, an important alternative source of information exists in the MIS. A search on the National Library of Medicine identifies more then 2,000 papers that have used MIS data in some capacity (search date 16–02-2022). These routine, large-scale household surveys are designed to produce snapshots of the malaria situation at national, regional and urban/rural levels. In Malawi, national MIS were conducted in 2010, 2012, 2014 and 2017 [25–27]. These MIS, together, capture the malaria situation and dynamics throughout the country and represent an essential source of information for policy development [1]. For example, MIS studies helped recognize the possible ineffectiveness of ITNs [10, 11], which led to the recent addition of pyrethroid-Piperonyl butoxide (PBO) nets to the vector control programme. The latest MIS survey was conducted in Malawi in April 2021. Data are currently being analysed and have not been released to the public.

Even with the recent addition of PBO and dual active ingredient nets to the vector control programme, traditional permethrin nets will likely remain an important part of control efforts in Malawi due to the uncertain durability of the next generation bed nets[28], their additional costs and unknown acceptance by the local population. In this study, the spatial relationship between ITN access and use, and malaria prevalence in children was investigated using MIS data from 2012, 2014 and 2017.

## Methods

### Country profile

Malawi is a southern African country with an estimated population of 18.6 million in 2019 [29]. The country consists of the Northern, Central and Southern regions, further divided into 28 districts. Malawi has three seasons, a rainy season from November to April, a cool-dry season from May to August and a hot-dry season from August to November. This sub-tropical climate is favourable for transmission of malaria. The main malaria vector, *Anopheles funestus*, is present throughout the country and throughout the year. The population along Lake Malawi and in the southern lowland are especially at high risk [15], as their environment is also ideal for the secondary malaria vectors *Anopheles gambiae *sensu stricto (*s.s.*) and *Anopheles arabiensis* [15, 30]. Even though *An. funestus* and *An. gambiae *sensu lato (*s.l.*) were susceptible to all insecticides in 2007, by 2010 pyrethroid resistance was found in both species [16, 31]. At present, it is suspected that pyrethroid and carbamate resistance is widespread across the country [32]. In 2007, the Malawian government started the distribution of LLINs. In 2012, 41% of the population owned at least one ITN with 29% sleeping under it the preceding night [25]. In the same year, the aim of universal bed net coverage (one LLIN per 1.8 people) was expressed. To achieve this, in 2014, 7 million nets were distributed throughout the country, further complemented by an additional 8 million in 2016 [1][1]. During all distribution campaigns hyperendemic and endemic areas were prioritized. To supplement the ITN campaigns, ITNs are also routinely distributed through the antenatal care service. Sporadic indoor residual spray (IRS) campaigns have also been conducted in industrial agricultural estates and in several districts [25, 34]. During the mass ITN distribution campaigns IRS districts do not receive ITNs. Although IRS use has been related to a reduction in parasitaemia in Malawi [35], due to their high cost and the spread of insecticide resistance, it was scaled back to Nkhotakota district in 2012 and implemented only sporadically until 2018. A timeline of the different malaria vector control activities in Malawi can be found in Additional file 1.

### MIS data

The MIS are designed to provide nationally, regionally and urban/rural representative data on 14 core malaria indicators during peak malaria transmission, including malaria prevalence and malaria control [36]. A different combination of households is surveyed each survey year using a two-stage stratified cluster design [37]. Field teams ask the head of household an array of questions. If the target respondent is unavailable, a person aged 15 or over living in each household is asked the questions. The field team also conduct malaria testing and geo-reference the location [38]. Household anonymity is maintained by displacing the coordinates randomly between 0 and 10 km within the second administrative boundary. The locations of the household clusters and their urban/rural classification are visualized in Additional file 2.

For this study, household-level MIS data from 2012, 2014 and 2017 were analysed with ‘household’ as the unit of analysis. The timing of the MIS in combination with the implementation of vector control activities in Malawi are tabulated in Additional file 1. A household is defined as one person or a group of people living together in a housing unit who acknowledge one adult as the head of the household [37]. The 2010 MIS was excluded, as it was not conducted by the DHS program, not geo-referenced and did not include Rapid Diagnostic Test (RDTs) results.

### Child malaria prevalence

In the MIS surveys, after verbal consent, children aged 6-to-59 months were tested for malaria using rapid diagnostic tests (RDTs) and microscopy of blood smears [38]. The RDT has a sensitivity of 99.5% and specificity of 98% for *Plasmodium falciparum* [39]. A small drop of blood from the finger or heel of a child was tested for *P. falciparum* in 2012 and 2014 using the SD Bioline rapid diagnostic test (Standard Diagnostic Inc., Korea) and in 2017 for *P. falciparum* and other *Plasmodium* species using the SD Bioline Malaria Ag P.f/Pan rapid diagnostic test (Standard Diagnostic Inc., Korea). The children who tested positive were offered malaria pharmacological treatment according to the current standard procedures for malaria treatment in Malawi [38]. A second blood sample was taken from the children for a thick and thin blood smear to confirm infection. The slides were checked in the laboratory for the presence of *Plasmodium* parasites by two independent microscopists. Both the RDT and blood smear results are shared in the MIS report. The NMCP in Malawi uses only the blood smear results for malaria prevalence calculations, while the RDT results are used for treatment of cases in the field. Correspondingly, the focus in this paper was on blood smear prevalence results.

### Insecticide-treated nets

The questionnaire respondents are asked to show the surveyor all the nets in the household. Surveyors are instructed to count and inspect these nets. From this ITN data, household ITN ownership (indicator 1: proportion of households with at least 1 ITN), household ITN access (indicator 2: proportion of households with at least 1 ITN for every 2 people sleeping in the house the night before), population ITN access (indicator 3: proportion of the population that could potentially sleep under an ITN the previous night, assuming each net could be used by 1.6 people (as suggested by [40])) and ITN use (indicator 4: proportion of the population that slept under an ITN the previous night) were calculated as proxies for ITN access and use [36]. A detailed description of the calculations is available in Additional file 3. These indicators were used to identify ownership and behavioural gaps. The ownership gap is the gap between household ITN ownership and population ITN access [36]. This gap highlights that although nets are reaching a large proportion of the population, the number of ITNs available in the householdis insufficient to cover everyone in the household (assuming one ITN provides protection for 1.6 people). The behavioural gap is the gap between population ITN access and ITN use [36]. This gap identifies if net access is sufficient to cover all people sleeping in the household (assuming one ITN provides protection for 1.6 people), yet ITNs are not being used.

### Statistical analysis

**Fig. 1:**
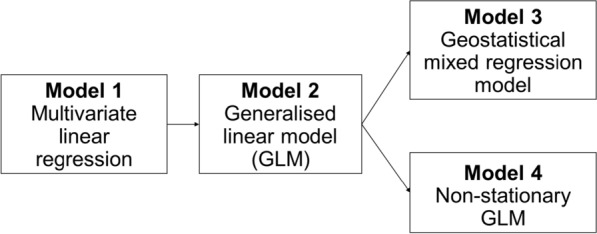
The four different model types used to investigate the relationship between ITN use and childhood malaria in Malawi

The data were analysed in the R software [41–43]. National averages and 95% confidence intervals were calculated for each variable in each survey separately using a weighting system that adjusts for selection probability differences [37]. Paired *t*-tests were undertaken to compare differences in malaria prevalence between RDTs and blood smear results. Four different model types were used to investigate the relationship between childhood malaria and ITN use in Malawi (Fig. 1). Two assumptions were made: (i) population ITN access is a good representation of the accessibility of good quality ITNs for the population in the area; and (ii) ITN use the previous night is a good representation of the overall use of ITNs by the population throughout the malaria transmission seasons due to the large sample size of the MIS.

An initial investigation was undertaken using the multivariate linear regression model (model 1). As the residuals were not normally distributed, the multivariate linear regression model was extended to a binomial generalized linear model (BGLM, model 2). In this model, variable selection was based on the Akaike information criterion (AIC). The selected predictor covariates consisted of the year of survey, urban/rural areas, population ITN access, ITN use and the interaction between population ITN access and ITN use, with malaria prevalence from blood smear used as the outcome variable. Residuals were mapped and tested for autocorrelation to check if values were independent.

#### Geostatistical mixed regression modelling

As the BGLM model (model 2) showed autocorrelation in the residuals, data were formally tested for spatial dependence in the variance. Spatial dependence was shown to be significant [see Additional file 4] and, therefore, spatial analysis was undertaken by fitting a Binomial generalized geostatistical linear model (BGGLM) [44] to the aggregated malaria prevalence data (model 3):

$$\mathrm{log}\left(\frac{p\left({x}_{i}\right)}{1-p\left({x}_{i}\right)}\right)=\,\mathrm{\alpha }+{\beta }_{1}{a}_{1}\left({x}_{i}\right)+{\beta }_{2}{a}_{2}\left({x}_{i}\right)+{\beta }_{3}{a}_{3}\left({x}_{i}\right)+{\beta }_{4}{a}_{4}\left({x}_{i}\right)+{\beta }_{5}{a}_{5}\left({x}_{i}\right)+S({x}_{i})+{Z}_{i}$$

where $\alpha$ is the intersect; ${\beta }_{1}$ the linear regression parameter for the survey year, ${\beta }_{2}$ the parameter for the rural/urban variable at location $i;$ ${\beta }_{3}$ the parameter for ITN population access; ${\beta }_{4}$ the parameter for ITN use; ${\beta }_{5}$ the parameter for the interaction between ITN population access and ITN use; $S\left({x}_{i}\right)$ a spatial Gaussian process; ${x}_{i}$ the *i*th spatial location and ${Z}_{i}$ the residual extra-binomial variation within a sampling location. The Monte Carlo maximum likelihood (MCML) estimation was used to fit the binomial model. The MCML estimation was repeated using the parameter values from the initial MCML to improve the MCML estimates [44].

There is a logical relation between the ITN population access and ITN use data variables, converging at 0 value. Further investigation of these variables indicated weak collinearity (variance inflation factor < 5), and adjustment in the models was not needed. However, as an interaction between both variables is expected, the interaction was left in the model.

As is common practice, a mixed model with a geostatistical spatial random effects term was adopted. This type of mixed model is justified where the residuals from the linear fixed effects term of the regression model are autocorrelated. The difference between urban and rural areas was accounted for by adding it as a covariate. To account for differences in data collection teams, malaria cycle periods and climate between the different survey years, the year was also included as a covariate.

#### Non-stationary generalized linear model

The BGGLM (model 3) is a global model in that its parameters are spatially stationary. To investigate the variation of parameters across space, a non-stationary GLM was fitted (model 4). This non-stationary method of regression considers only a portion of the data around the prediction point at a time, and is sometimes referred to as ‘local mapping.’ A so-called ‘kernel’ is moved across the study area and at each location in a pre-defined grid, the regression coefficients are estimated with the GLM (model 2) as its base, using the data weighted by the kernel. The size of kernel can be adaptive, to ensure that sufficient information is included even in areas where data are sparse. In this way, the non-stationary method allows the estimated regression parameters to be mapped throughout the study area [45]. Specifically, it calculates local regression parameters by fitting a regression model for malaria prevalence $p$ such that:

$${p}_{({u}_{i},{v}_{i})}={\alpha }_{i}+ \sum_{k=1}^{m}{\beta }_{k}({u}_{i},{v}_{i}){x}_{k,i}+{Z}_{i}$$

where ($${u}_{i}$$,$${v}_{i})$$ represents the coordinates at location $$i$$; $${\alpha }_{i}$$ the intersect at location $$i$$;$$m$$ the number of independent variables; $$\beta {(u}_{i}$$,$${v}_{i})$$ the local regression parameter for the $$k$$ th independent variable at location $$i$$; $${x}_{k,i}$$ the $$k$$ th covariate; and $${Z}_{i}$$ the error at location $$i$$. This expression allows the $$\beta$$ to vary with the location coordinates ($${u}_{i}$$,$${v}_{i})$$, making the model spatially non-stationary; and allows $$\beta$$ to be estimated via weighted least squares with the weights matrix obtained from a Gaussian kernel, attributing larger weights to values of predictors from more proximate locations. Analysis was undertaken using the package 'GWmodel' [46]. Standard errors were estimated using the bootstrap function, with coefficients re-estimated 1,000 times at each grid point. The size of the kernel is controlled by the bandwidth, identified using both the cross-validation and AIC corrected (AICc) approach within the package. Collinearity is minimized as described by the R package documentation.

## Results

### Exploratory analysis

The MIS data used for this research consisted of 10,538 households in 430 different locations collected during three different years (Table 1). Malaria prevalence calculated using RDT results was higher than when calculated using blood smears for 2012 (paired *t*-test *P* < 0.001, df = 139), 2014 (*P* = 0.012, df = 139) and 2017 (*P* < 0.001, df = 149). A comparison of malaria prevalence for both RDTs and blood smears on the same children showed more than 80% overlap [see Additional file 5].

**Table 1:** Summary of Malawi MIS data from 2012, 2014 and 2017

	2012	2014	2017
Survey months	March–April	May–June	April–June
Households	3404	3405	3729
Clusters	140	140	150
Average number of household members in household (95% CI)	4.17 (4.07–4.26)	4.12 (3.99–4.25)	4.46 (4.38–4.55)
Malaria prevalence (%)			
RDT	42.5 (37.0–47.9)	36.2 (29.4–43.0)	36.3 (31.7–40.8)
Blood smear	27.1 (23.1–31.2)	32.1 (25.5–38.7)	23.9 (20.3–27.4)
ITN data			
Total nets	3316	4754	7182
Total ITNs	2932	4562	6752
Household ownership (%)	55 (51.2–58.9)	70.2 (65.8–74.6)	82.1 (80.0–84.2)
Household access (%)	19 (15.6–22.3)	30.3 (26.8–33.8)	41.7 (39.1–44.4)
Population access (%)	33.1 (29.8–36.4)	47.1 (43.4–50.8)	58.4 (56.2–60.6)
ITN use (%)	41.1 (37.3–44.9)	53.1 (49.3–56.9)	57.4 (55.0–59.9)

Averages and proportions are calculated using the Demographic and Health Surveys weighting scheme [37]. Unsuccessful tests were noted as ‘other’. Unsuccessful tests were ‘inconsistent data’, ‘not present’, ‘refused’, ‘sample not found’ or ‘test undetermined’

**Fig. 2:**
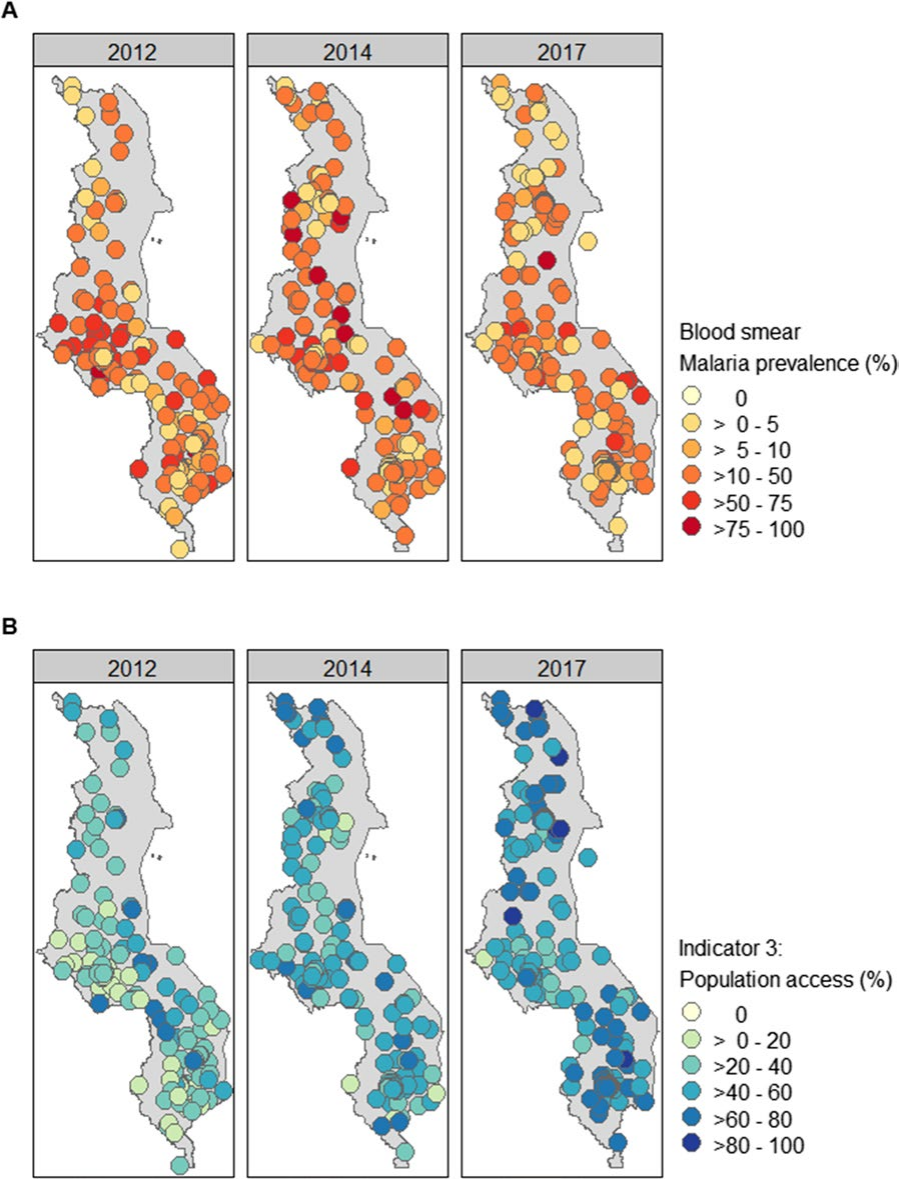
Malaria prevalence and ITN access summary results for the household clusters surveyed in 2012, 2014 and 2017. **A** malaria prevalence in children measured using blood smear test in the different household clusters. **B** Population ITN access in the different household clusters with the assumption that one ITN provides protection for 1.6 people

Although malaria prevalence in the different survey years did not differ (Table 1), the number of positive cases decreased in central Malawi (Fig. 2). Malaria prevalence was lower in urban areas compared to rural areas (both RDT and blood smear P < 0.001) [see Additional file 6].

**Fig. 3:**
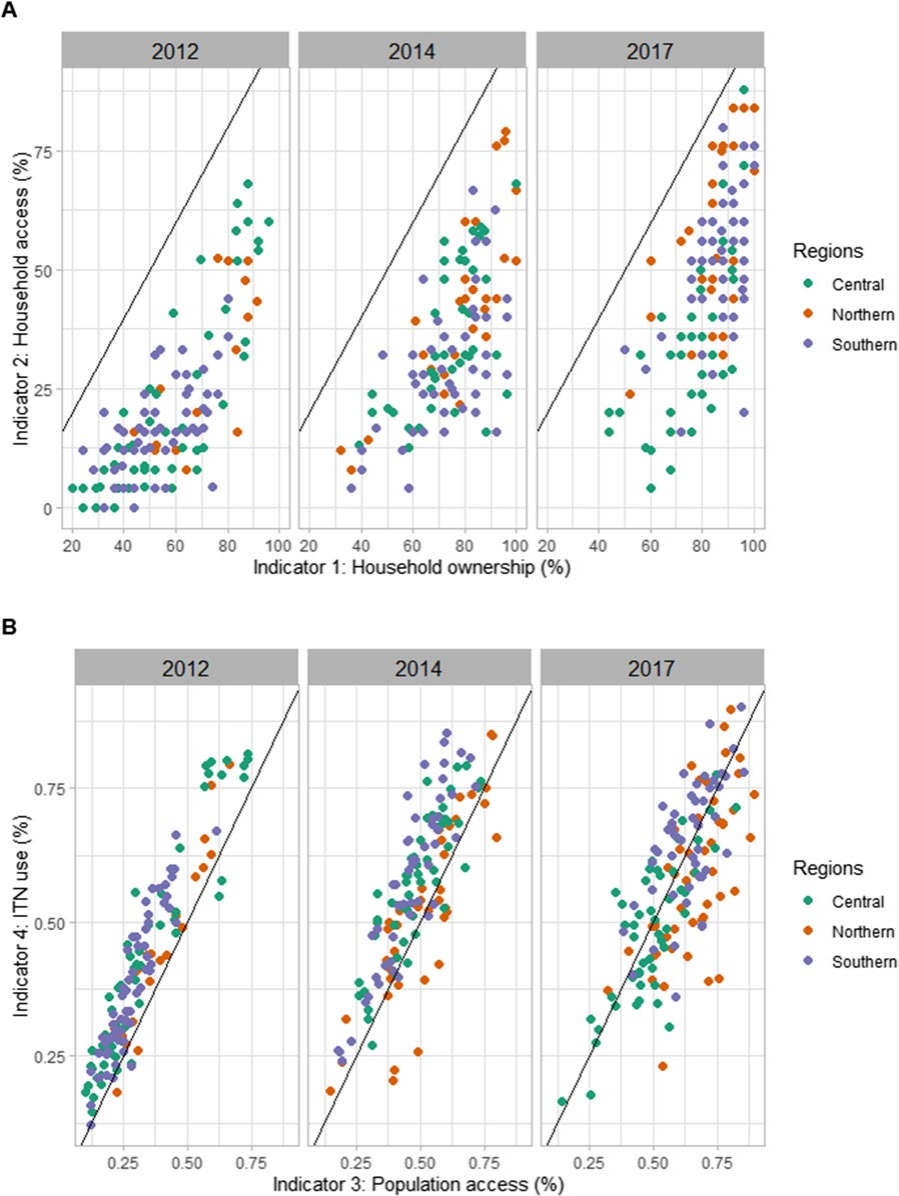
Scatterplots visualizing the ITN ownership gap and behavioural gap in 2012, 2014 and 2017, with colours indicating the different regions. The black lines are the trend lines representing no gap. **A** Comparison of household ITN ownership to household ITN access to visualize the ITN ownership gap. **B** Comparison of population ITN access to ITN use to visualize the behavioural gap

All ITN indicators suggested an increase in ITN access and use through time (Table 1). By 2017, household ownership had increased from 55 to 82%. Ownership was high throughout the country, with most households owning at least one ITN (Fig. 2) [see Additional file 7]. However, this high household ownership did not translate into similarly high household access. In 2017, only 42% of households had sufficient ITNs to cover all sleeping people and ITN access for the population was below 60% for the majority of areas surveyed. Net coverage in central Malawi was slightly lower than for the rest of the country, with also fewer people sleeping under an ITN [see Additional file 7]. There is evidence of an ownership gap, with ITN campaigns reaching most households across the country, but not supplying sufficient numbers for complete coverage (Fig. 3). There is no evidence of a behavioural gap in 2017, with the increase in population ITN access from 2012 to 2017 aligned with an increase in ITN use.

#### Bionomial generalized geostatistical linear mixed regression modelling

The BGGLM revealed a negative association between malaria prevalence and ITN population access in all regions in Malawi, with low child malaria prevalence occurring in areas with high population access to nets. In contrast, malaria prevalence was positively associated with sleeping under a net, with high use occurring in areas with high child malaria prevalence (Table 2). Both ITN variables showed heterogeneity across the different regions, suggesting a heterogeneous relationship between child malaria prevalence and ITN population access and ITN use. The interaction between ITN population access and ITN use was not significant.

**Table 2:** Results of binomial geostatistical mixed regression of blood smear malaria prevalence against selected covariates

	ITN population access^a^		ITN use^a^		Rural		year 2014		year 2017		Interaction ITN population access and ITN use	
Areas	OR	*P*-value	OR	*P*-value	OR	*P*-value	OR	*P*-value	OR	*P*-value	OR	*P*-value
Malawi	0.01 (0.008–0.06)	< 0.001*	6.80 (1.38–33.43)	0.018*	3.35 (2.45–4.57)	< 0.001*	1.34 (1.09–1.65)	< 0.001*	1.09 (0.84–1.42)	0.504	4.40 (0.37–52.01)	0.239
north	0.02 (0.001–0.14)	< 0.01*	12.19 (0.32–58.22)	0.035*	1.70 (0.92–3.41)	0.0822	1.79 (1.09–2.93)	0.020*	0.90 0.48–1.69	0.752	3.99 (0.05–21.96)	0.677
central	0.07 (0.012–0.45)	< 0.001*	6.43 (1.05–39.19)	0.034*	3.67 (2.23–6.04)	< 0.001*	1.37 (1.12–1.68)	0.0019*	0.97 (0.75–1.25)	0.835	1.38 (0.11–16.70)	0.750
south	0.05 (0.0004–0.68)	0.002*	3.27 (0.06–75.16)	0.558	4.03 (2.09–7.79)	< 0.001*	1.67 (0.94–2.97)	0.078	2.07 (0.91–4.72)	0.082	12.57 (0.19–59.21)	0.137

Odds ratio (OR) and the 95% confidence intervals (95%CI), calculated from the parameter estimates and the associated standard errors fitted using Monte Carlo maximum likelihood estimation

*Indicates significant difference (P < 0.05)

^a^ITN population access ranged from 0 to 1 proportion of the population having access to ITNs and ITN use ranged from 0 to 1 proportion of people sleeping under a net

There was a clear relationship between malaria prevalence and the urban/rural variable, with child malaria cases three times higher in rural areas than urban areas. The same model, run independently for each region, indicated that the difference in malaria prevalence between urban and rural areas was demonstrated mainly in central and southern Malawi, where malaria was four times higher in rural areas (Table 2). Child malaria prevalence was lower in 2012 than 2014, but did not differ between 2012 and 2017.

#### Non-stationary spatial generalized linear model

The non-stationary coefficient estimates for both ITN population access and ITN use for the three different years were mapped [see Additional file 8]. The parameters (i.e., relationship) between child malaria prevalence and the indicators exhibit some geographical variation, although the standard errors are large (Table 3). The north of the country shows parameters consistently significant compared to the south of the country. A temporal difference appears between 2014 and 2017, with the correlation between child malaria prevalence and both indicators reduced in 2017 compared to 2014.

**Table 3:** Results of the non-stationary generalized linear model of blood smear malaria prevalence against ITN population access and ITN use in the different survey years

	Survey year	Global $\beta$ estimate (se)	Range	Global OR (95%CI)	Range	*P*-value
ITN population access	2012	− 0.98 (0.32)	− 1.29 to − 0.35	0.38 (0.20–0.70)	0.28 to 0.70	< 0.01*
	2014	− 0.93 (0.24)	− 1.29 to − 0.55	0.39 (0.25–0.63)	0.28 to 0.58	< 0.001*
	2017	− 0.37 (0.13)	− 0.47 to − 0.11	0.69 (0.53–0.89)	0.63 to 0.90	< 0.01*
ITN use	2012	0.68 (0.28)	0.25 to 0.88	1.97 (1.14–3.42)	1.28 to 2.41	0.0168 *
	2014	0.76 (0.21)	0.56 to 1.22	2.14 (1.42–3.23)	1.75 to 3.39	< 0.001*
	2017	0.37 (0.12)	0.03 to 0.45	1.45 (1.14–1.83)	1.03 to 1.57	< 0.01*

Global parameter estimates and the associated standard errors (se) are shown with its range across the country. Additionally, the odds ratio (OR) and the 95% confidence intervals are presented

*Indicates significant difference (P < 0.05)

## Discussion

This study presents an important, key modelling approach that allows malaria control programmes to spatially unravel the relationship between child malaria prevalence and ITN distribution. The non-stationary generalized linear model helped visualize variation in the relationship between malaria cases and bed net indicators. Increasing household access to and ownership of ITNs resulted in a decrease in malaria cases. However, this did not occur homogeneously across the country. The ITN coverage and use increased from 2012 to 2017, while child malaria prevalence decreased only in some areas. The BGGLM regression using MIS survey data showed that child malaria prevalence had a negative association with ITN population access and a positive association with ITN use. However, it is challenging to identify cause and effect without temporally linked data. The MIS data are currently the best information available to understand the impact of ITNs on malaria prevalence in Malawi. The large variation in odds ratio reflects the high uncertainty in the data and the need for further localized data that takes into account any potential confounder, to spatially disentangle the relationship between ITN indicators and malaria prevalence.

A negative association between malaria prevalence and ITN population access in all regions of Malawi was found. Areas with high population access to nets had lower malaria prevalence than areas with low access. This corresponds to earlier work in Malawi, where ITN ownership was found to be protective against malaria parasitaemia in children [7, 47]. The non-stationary spatial analysis highlighted heterogeneity in the relationship with some areas showing no negative association. The assumption was made that nets maintained physical integrity and bioefficacy of insecticides at least one year post-distribution. Yet, the lack of a discernible relationship could indicate that the roll out was slow, new nets from the campaigns were not used (still in packaging), nets were not used consistently or nets were ineffective. The nets are unlikely to be entirely ineffective, as even with high insecticide resistance the physical barrier of nets and sublethal effect of the insecticides results in a negative relationship between malaria prevalence and ITNs [47–49]. It is likely that other factors also affected prevalence, creating artefacts in the relationship between the outcome and predictor. For example, behavioural resistance, with mosquitoes biting when people are not protected by the nets, could play a role [50, 51]. The quality of the nets has also not been considered, while studies have shown that using a mosquito bed net that is more than one year old is a risk factor for malaria in Malawi [52]. Moreover, extreme droughts the year prior could have impacted overall malaria prevalence dynamics as the model does not account for key environmental factors [53].

There was a positive relationship between malaria prevalence and ITN use in all Malawian regions, with high net use occurring in areas with high child malaria prevalence. The question ‘did you sleep under a bed net last night?’ was used as a proxy for overall ITN use. The question focuses on ‘last night’ and does not capture the overall ITN use behaviour. The positive association found here between malaria prevalence and net use is likely due to the large numbers of malaria cases motivating the population to sleep under nets more regularly in the short term. The MIS household surveys are sensitive to recall bias and social desirability factors [54]. Additionally, nightly variables, such as temperature and mosquito density, could also greatly impact the use of nets [55]. For ITN use throughout the malaria season to be related to malaria prevalence, thus identifying its protective ability, temporal studies are necessary where fieldworkers independently confirm bed net use over both space and time throughout the season.

This study revealed spatial heterogeneity in the relationship between malaria prevalence and the ITN indicators, with child malaria prevalence higher in rural than urban areas. It is widely accepted that malaria dynamics differ between rural and urban areas due to differences in demographics, socioeconomics, housing, drainage and access to health care [22, 56–59]. Generally, in Malawi malaria is more prevalent in rural areas, although high case numbers have also been reported in urban areas [10, 11, 22, 56, 58, 60]. Malaria control likely has a different impact in the urban and rural habitats, due to environmental differences and population movements. Furthermore, the dichotomization of the factors areinconsistent across the research field (the definition of what an urban or rural area is differs), with a more nuanced definition of urban malaria risk and prevention efforts necessary in Malawi to control adequately for the contextual factors that drive malaria prevalence [61]. The results from this study further substantiate the complexity of urban/rural malaria dynamics [22, 56] and show the importance of including this factor in the analysis to investigate the impact of vector control for sustainable intervention measures.

Many studies have shown that the scale-up of ITNs has protected the Malawi population from malaria [6–9]. This study shows that this relationship varies across space. Heterogeneity in malaria cases has been identified previously in Malawi [14, 52, 62]. Malaria epidemiology is a complex dynamic between many factors at the individual, household and community levels [62]. Confounders can have a large impact on this heterogeneity, and they are challenging to identify. The introduction of vector control in this environment can have very different outcomes, with the decline rate in malaria prevalence known to be very different across the country [14]. One possible explanation for spatial heterogeneity in the relationship between malaria prevalence and ITN use is that it captures the heterogeneity in malaria prevalence, with net coverage homogeneously high throughout the country. A small group of households can account for a majority of cases, which results in spatial heterogeneity in even small geographical areas [63]. Another possibility is that insecticide and behavioural resistance of vector species is impacting net efficacy. Little information about the spatial distribution of malaria vectors and their resistance status is available, but reports do indicate heterogeneity in its spread with high resistance reported in the south and around Lake Malawi [3]. It is also important to note the potential effect of other malaria control interventions on the heterogeneity of the relationship between malaria prevalence and ITNs, including the use of IRS, larval source management and house improvements [34, 64] on malaria prevalence, which have not been considered in this study. Understanding the spatial dynamics will help increase the effectiveness of vector control campaigns, for example, by changing to bed nets that kill resistant mosquitoes more effectively or shifting to different control tools altogether in specific areas. The fine-scale spatial and temporal heterogeneity and their causes need to be investigated further by implementing field studies designed to answer these specific questions.

In concurrence with the recommendations from the Ministry of Health in Malawi, and in alignment with prior Malawian MIS studies [10, 11], blood smear data were used as the sole proxy for malaria prevalence. Both the RDT and blood smear malaria testing methods have strengths and limitations [65]. As shown here, the RDT generally produces higher positivity rates than blood smears, as it measures antigens that are detectable in the blood up to four weeks after parasite clearance [66]. This is contrary to blood smear tests, which measure the physical presence of malaria parasites. From a modelling perspective, instead of choosing one indicator, joint distribution modelling of RDT and blood smear results could help improve inference. Recently, Amoah et al.[67] developed a geostatistical framework to combine spatially referenced disease prevalence data from multiple diagnostics. Joint distribution models draw benefit from the combination of different diagnostics, although particular care needs to be taken for diagnostics with large discrepancies in sensitivity and specificity.

It is important to note that malaria prevalence is not the only way to measure malaria burden in a country. Studies have found mortality reduction without a decrease in malaria prevalence, and the other way around [68]. Furthermore, malaria prevalence here is focused on children aged 6-to-59 months, while school-aged children are both at higher risk of infection and asymptomatic infection [69, 70]. The study could, thus, be improved by using malaria prevalence in the entire population as an outcome variable, which is not possible with MIS data. Excluding this important risk group from the analysis could have skewed the data and results. Additionally, the MIS data are a snapshot and do not include the seasonality of malaria. Although surveys are planned during peak malaria season, as shown by Chirombo et al.[15], this peak differs yearly in Malawi. Whether data are collected during the peak season or two weeks later hugely impacts malaria prevalence estimates. If snapshots of malaria prevalence are compared between the different years without a clear understanding of the seasonal dynamics in these different years, this will greatly influence the analysis and incorrect conclusions can be made. It is important to place malaria prevalence from MIS data in the context of the country and investigate how this is linked to mortality and other malaria indicators.

The MIS data are currently the only data available in Malawi to investigate the relationship between malaria prevalence and ITN use. As the MIS has not been designed for this purpose, caution is advised when interpreting the results. Studies designed to investigate the impact of ITNs are indispensable to understand the efficacy of ITNs [68]. Until these studies are available, creative solutions are necessary to analyse MIS data. Two studies have previously used MIS data from 2012 and 2014 to investigate the relationship between child malaria prevalence and ITN use. Contrary to this study, both found that the number of bed nets per household was not significantly associated with malaria morbidity [10, 11]. Both studies focused on socio-demographic characteristics, while this study included the spatial coordinates of the data within the analysis. This study shows that the impact of ITNs differs geographically. For vector control programmes to make informed decisions about future control activities, it is essential to have access to both country-wide and spatially disaggregated analyses of ITNs impact. This spatial disaggregation is especially important with an increase in IRS activities since 2019 and a combination of PBO and dual active ingredient bed nets distributed during mass ITN campaigns in 2018 and 2021 [33].

The non-stationary generalized linear model visualized the geographically changing relation between malaria prevalence and the ITN indicators. Although the geostatistical mixed regression model presented the overall spatial relationship between child malaria prevalence and bednet indicators, it did not allow for visualization of variation of these relationships across Malawi. The maps produced by the non-stationary model can help vector control programme spatially disentangle the impact of interventions on malaria prevalence for vector control programs. The non-stationary model has some limitations. For example, the bandwidth is optimized based on accurate prediction of the response variable, not on accurate estimation of the coefficients [46]. Especially when the regression model is fitted within a small kernel or with limited data, collinearity can be a problem [71]. Nevertheless, for spatially clustered data such as the MIS data, this method can be appropriate to provide insights into how estimated relations vary across the country. Although relationships are not causal, they could highlight underlying covariates that have been left unmeasured. It is an important tool for eco-epidemiological studies [72–74], especially for a disease such as malaria, that is so closely related to the environment and the sociodemographic dynamics of the population. Yet, spatially non-stationary models have only rarely been used in malaria research.

## Conclusion

Malaria prevalence in children under five had a negative association with ITN population access and a positive association with ITN use in unadjusted models, showing spatial heterogeneity in these relationships across Malawi. The non-stationary generalized linear model is an important modelling approach that helps vector control programmes visualize variation in the relationship between malaria cases and intervention methods. This study highlights the complexity of the relationship between malaria and ITNs and the clear need for spatially disaggregated data and models to inform localized control activities.

## Supplementary Information

**Additional file 1.** Timeline of malaria indicator surveys (MIS) in combination with the main vector control activities implemented in Malawi from 2011 to 2017.

**Additional file 2.** Household cluster location for each Malaria Indicator Survey (MIS) round.

**Additional file 3.** Description of the insecticide-treated net indicator calculations.

**Additional file 4.** Variogram showing spatial dependence in the variance.

**Additional file 5.** Comparison of malaria prevalence calculated using RDT and blood smear results.

**Additional file 6.** Blood smear malaria prevalence in urban and rural areas for 2012, 2014 and 2017 with median.

**Additional file 7.** Insecticide treated net indicators mapped for 2012, 2014 and 2017.

**Additional file 8.** The geographically weighted regression analysis results with β the estimated coefficient range and the standard error (se).

## Data Availability

The datasets analysed during the current study are available in the DHS program repository, which can be accessed through (https://dhsprogram.com/).
