# Supplementary material for: Unravelling the impact of insecticide-treated bed nets on childhood malaria in Malawi
Source: Malar J. 2023 Jan 13;22:16. doi: 10.1186/s12936-023-04448-y (PMC9837906; doi:10.1186/s12936-023-04448-y)
Supplement: Supplementary file 1 — Additional file 1. Timeline of malaria indicator surveys (MIS) in combination with the main vector control activities implemented in Malawi from 2011 to 2017. [file 12936_2023_4448_MOESM1_ESM.docx]

# Supplementary information 1

*Timeline of malaria indicator surveys (MIS) in combination with the main vector control activities implemented in Malawi from 2011 to 2017.*
