# Supplementary material for: Unravelling the impact of insecticide-treated bed nets on childhood malaria in Malawi
Source: Malar J. 2023 Jan 13;22:16. doi: 10.1186/s12936-023-04448-y (PMC9837906; doi:10.1186/s12936-023-04448-y)
Supplement: Supplementary file 2 — Additional file 2. Household cluster location for each Malaria Indicator Survey (MIS) round. [file 12936_2023_4448_MOESM2_ESM.docx]

# Supplementary information 2

| 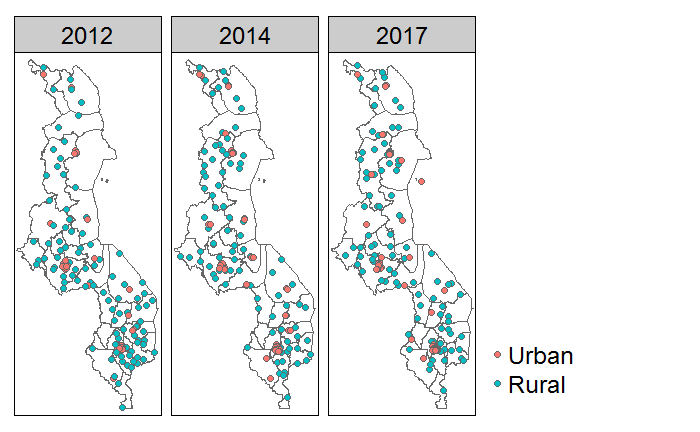 |
| --- |
| *Household cluster for each MIS round, with colours indicating household locations in urban or rural area.* |
