# Supplementary material for: Unravelling the impact of insecticide-treated bed nets on childhood malaria in Malawi
Source: Malar J. 2023 Jan 13;22:16. doi: 10.1186/s12936-023-04448-y (PMC9837906; doi:10.1186/s12936-023-04448-y)
Supplement: Supplementary file 3 — Additional file 3. Description of the insecticide-treated net indicator calculations. [file 12936_2023_4448_MOESM3_ESM.docx]

# Supplementary information 3

Detailed description of the insecticide-treated net calculations

| indicator | Unit of analysis | Calculation |
| --- | --- | --- |
| Household ownership | Cluster of households | $\hat{p}_{h}$ $= \frac{x_{b}}{x_{t}}$  $\hat{p}_{h}$ = household ownership  $x_{b}$ = sum of households with at least one ITN  $x_{t}$ = sum of households surveyed |
| Household access | Cluster of households | Step 1  $\hat{p}_{p}= \frac{x_{c}}{x_{t}}$  $\hat{p}_{p}$ = proportion of population with access to 1 ITNs per 2 people  $x_{c}$ = total number of ITNs in household  $x_{t}$ = total number of household members that slept in the household the previous night, including visitors  Step 2  $\hat{p}_{p}$ < 0.5, $x_{d}$ is 0  $\hat{p}_{p}$ ≥ 0.5, $x_{d}$ is 1  $\hat{p}_{p}$ = proportion of population with access to 1 ITNs per 2 people  $x_{d}$ = households with full household access to ITNs, excluding households without a sleeping person  Step 3  $\hat{p}_{a}$ $= \frac{{sum(x}_{d})}{x_{u}}$  $\hat{p}_{a}$ = household access  $x_{d}$ = households with full household access to ITNs  $x_{u}$ = total number of households surveyed in the cluster, excluding households without a sleeping person |
| Population access | population | Step 1  $x_{e}$ > $x_{t}$, adjust $x_{e}$ = $x_{t}$  $x_{e}$ = Potential ITN user, calculated by total number of ITNs * 1.64, with the assumption that one ITN provided protection for 1.64 people [55]  $x_{t}$ = total number of household members that slept in the household the previous night, including visitors  Step 2  $\hat{p}_{q}= \frac{sum(x_{e})}{x_{t}}$  $\hat{p}_{q}$ = population access  $x_{e}$ = Potential ITN user  $x_{t}$ = total number of household members that slept in the household the previous night, including visitors |
| ITN use | population | $\hat{p}_{r}$ $= \frac{x_{g}}{x_{t}}$  $\hat{p}_{r}$ = ITN use  $x_{g}$ = sum of number of people who slept under an ITN the night before, sum(hml11_1 to hml11_7)  $x_{t}$ = total number of household members that slept in the household the previous night, including visitors (*hv013*) |
