# Supplementary material for: Unravelling the impact of insecticide-treated bed nets on childhood malaria in Malawi
Source: Malar J. 2023 Jan 13;22:16. doi: 10.1186/s12936-023-04448-y (PMC9837906; doi:10.1186/s12936-023-04448-y)
Supplement: Supplementary file 4 — Additional file 4. Variogram showing spatial dependence in the variance. [file 12936_2023_4448_MOESM4_ESM.docx]

# Supplementary information 4

| 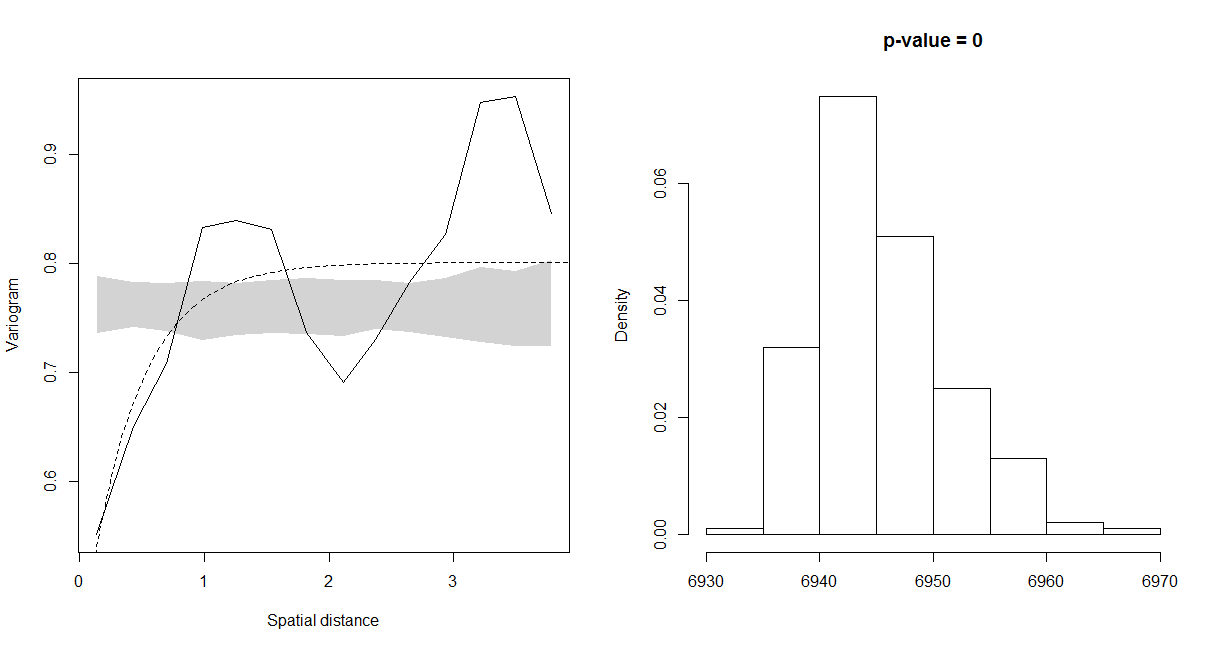 |
| --- |
| *Spatial dependence in the variance is shown by the variogram. The grey shaded areas are the Monte Carlo envelopes of the simulated variance, which is obtained by randomly assigning childhood malaria prevalence values at the different geographical locations and calculating the empirical variogram.* |
