# Supplementary figures and images for: Unravelling the impact of insecticide-treated bed nets on childhood malaria in Malawi
Source: Malar J. 2023 Jan 13;22:16. doi: 10.1186/s12936-023-04448-y (PMC9837906; doi:10.1186/s12936-023-04448-y)

# Supplementary information 5

| 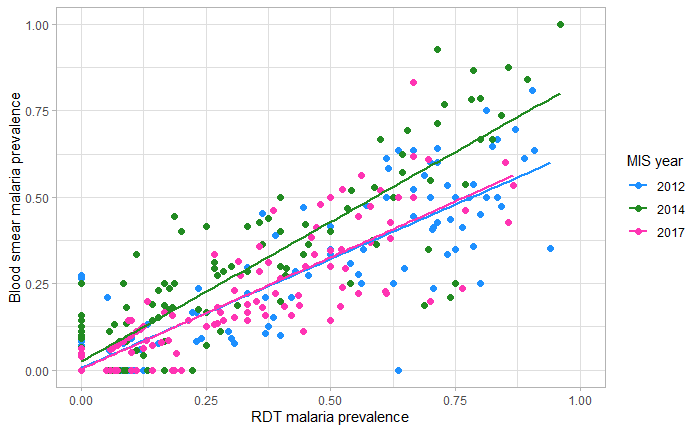 |
| --- |
| *Malaria prevalence comparison between RDT and blood smears.* |

Supplement: Supplementary file 5 — Additional file 5. Comparison of malaria prevalence calculated using RDT and blood smear results. [file 12936_2023_4448_MOESM5_ESM.docx]
