# Supplementary material for: Unravelling the impact of insecticide-treated bed nets on childhood malaria in Malawi
Source: Malar J. 2023 Jan 13;22:16. doi: 10.1186/s12936-023-04448-y (PMC9837906; doi:10.1186/s12936-023-04448-y)
Supplement: Supplementary file 6 — Additional file 6. Blood smear malaria prevalence in urban and rural areas for 2012, 2014 and 2017 with median. [file 12936_2023_4448_MOESM6_ESM.docx]

# Supplementary information 6

| 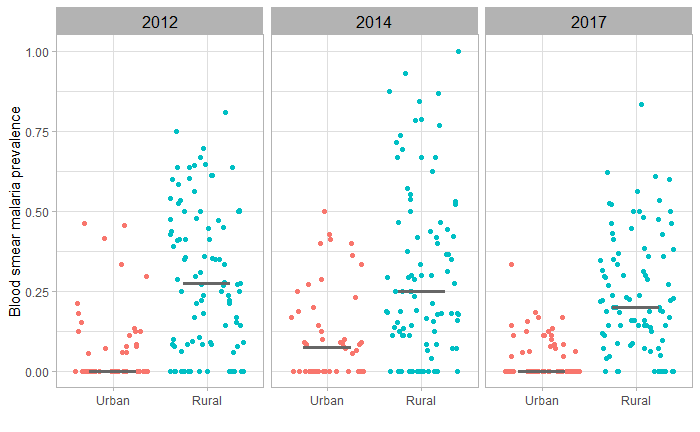 |
| --- |
| *Blood smear malaria prevalence in urban and rural areas for 2012, 2014 and 2017 with median.* |
