# Supplementary material for: Unravelling the impact of insecticide-treated bed nets on childhood malaria in Malawi
Source: Malar J. 2023 Jan 13;22:16. doi: 10.1186/s12936-023-04448-y (PMC9837906; doi:10.1186/s12936-023-04448-y)
Supplement: Supplementary file 7 — Additional file 7. Insecticide treated net indicators mapped for 2012, 2014 and 2017. [file 12936_2023_4448_MOESM7_ESM.docx]

# Supplementary information 7

| **A**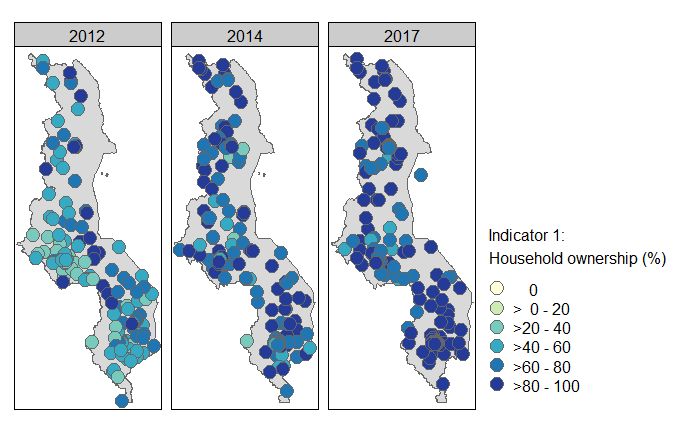 |
| --- |
| 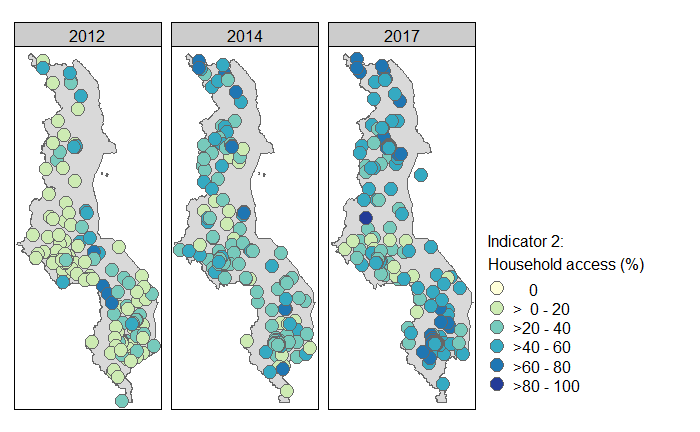**B** |
| **C**  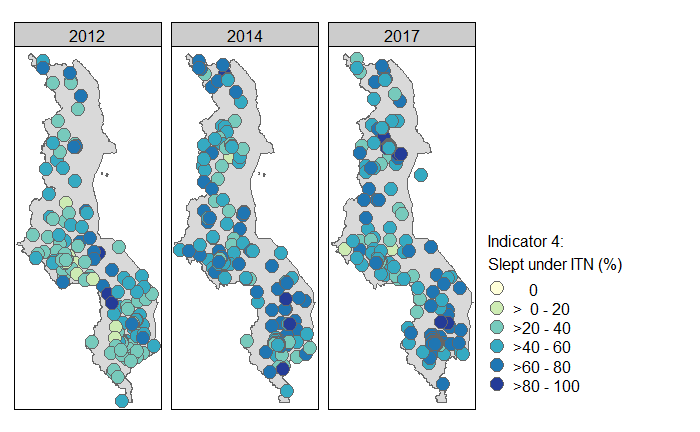 |
| *Insecticide treated net indicators from 2012, 2014 and 2017. A) Household ownership, B) household access, C) population that slept under an insecticide treated net (ITN) the night before the survey.* |
