# Supplementary material for: Unravelling the impact of insecticide-treated bed nets on childhood malaria in Malawi
Source: Malar J. 2023 Jan 13;22:16. doi: 10.1186/s12936-023-04448-y (PMC9837906; doi:10.1186/s12936-023-04448-y)
Supplement: Supplementary file 8 — Additional file 8. The geographically weighted regression analysis results with β the estimated coefficient range and the standard error (se). [file 12936_2023_4448_MOESM8_ESM.docx]

# Supplementary information 8

| **Year** | **Population access** | **ITN use** | |  | |
| --- | --- | --- | --- | --- | --- |
| **2012** | 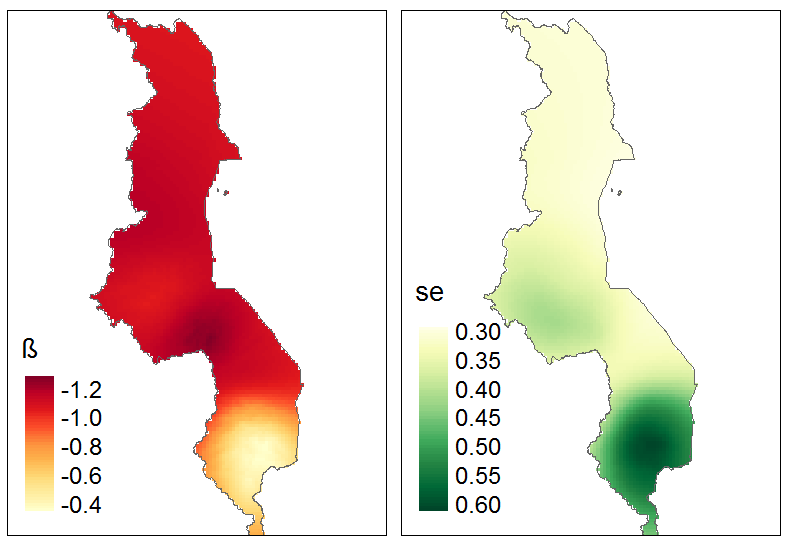 | 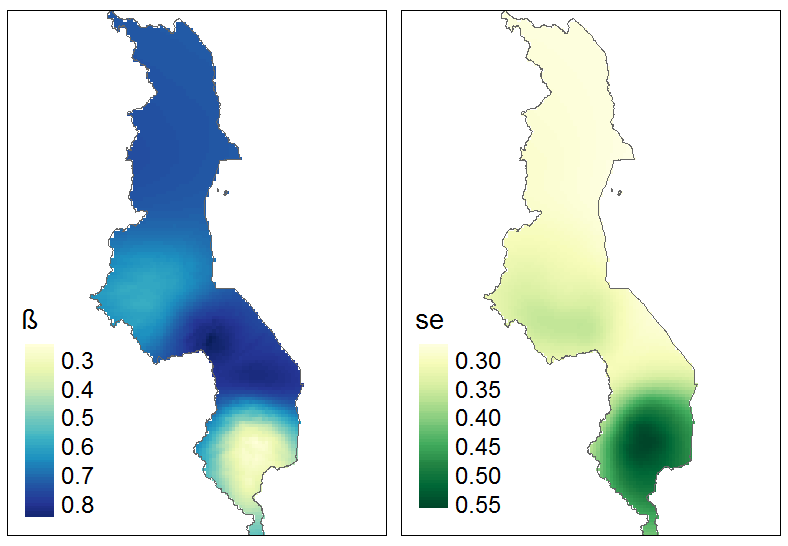 |  | |  |
| **2014** | 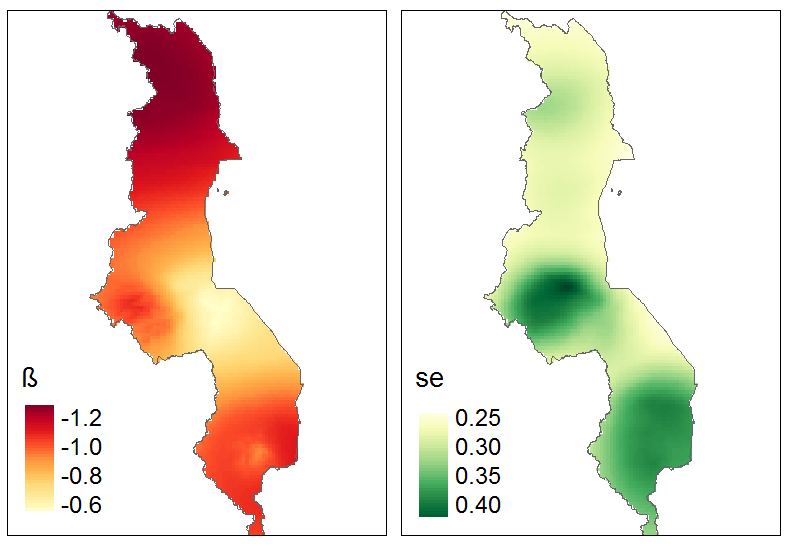 | 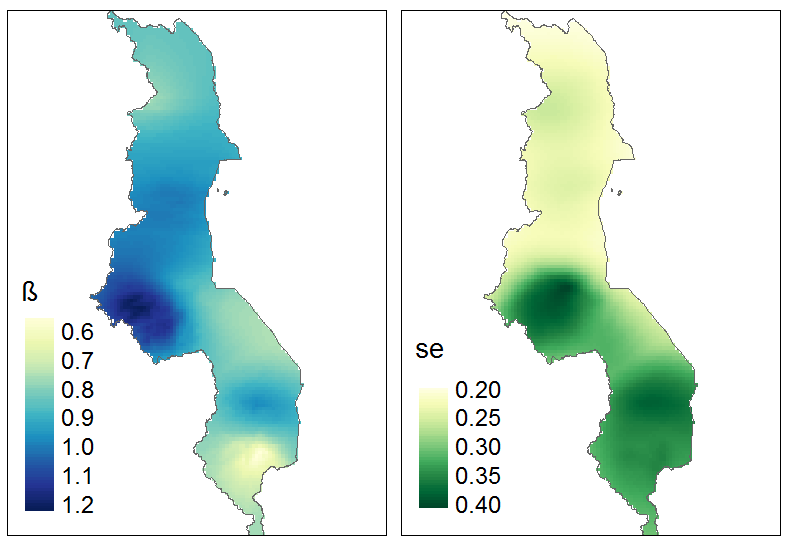 |  | |  |
| **2017** | 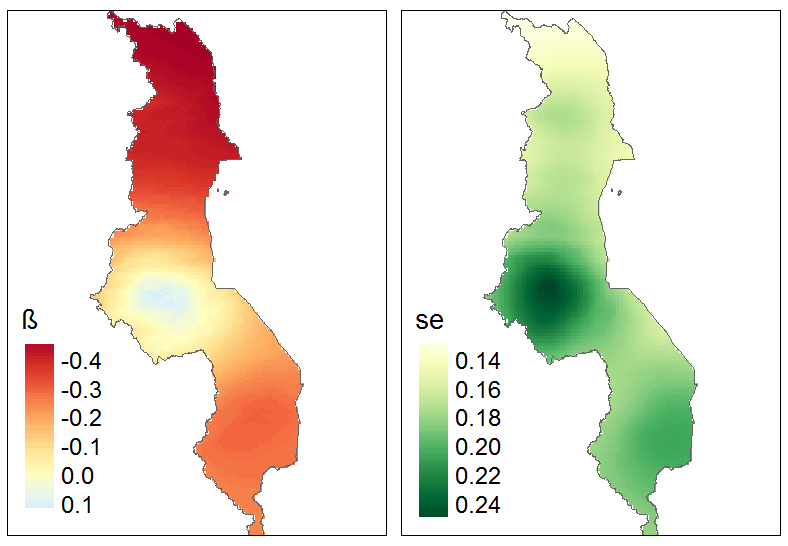 | 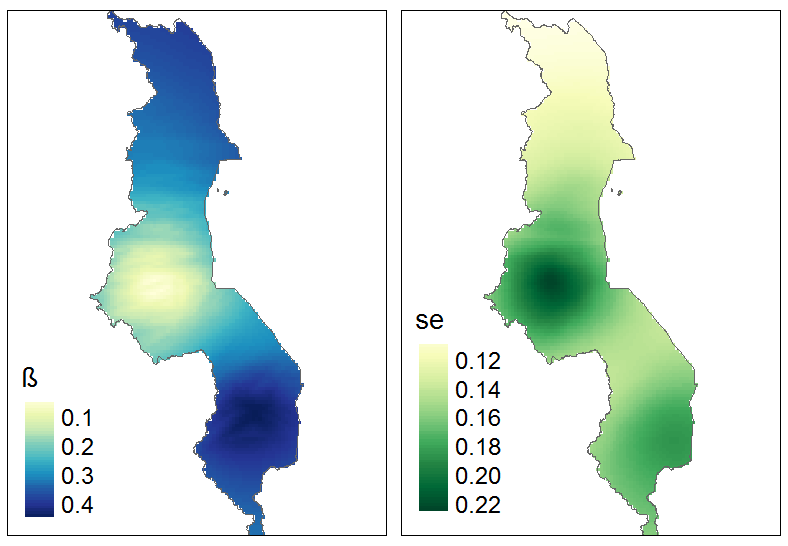 |  | |  |
| *The geographically weighted regression analysis results with β the estimated coefficient range and the standard error (se).* | | | |  | |
